# Discordant NIPT result in a viable trisomy‐21 pregnancy due to prolonged contribution to cfDNA by a demised trisomy‐14 cotwin

**DOI:** 10.1002/ccr3.1424

**Published:** 2018-03-07

**Authors:** Ron Hochstenbach, Martin G. Elferink, Patrick H. A. van Zon, Klaske D. Lichtenbelt, Jeske van Harssel, Heleen Schuring‐Blom, Godelieve C.M.L. Page‐Christiaens

**Affiliations:** ^1^ Department of Genetics University Medical Centre Utrecht Utrecht The Netherlands; ^2^ Department of Obstetrics and Gynecology University Medical Centre Utrecht Utrecht The Netherlands

**Keywords:** Cell‐free DNA, false‐positive result, noninvasive prenatal testing, vanishing twin

## Abstract

One of the confounders in noninvasive prenatal testing (NIPT) is the vanishing twin phenomenon. Prolonged contribution to the maternal Cell‐free DNA (cfDNA) pool by cytotrophoblasts representing a demised, aneuploid cotwin may lead to a false‐positive outcome for a normal, viable twin. We show that a vanishing trisomy‐14 twin contributes to cfDNA for more than 2 weeks after demise.

## What is Already Known About this Topic?

- Noninvasive prenatal testing for fetal aneuploidies can result in discordant results when an aneuploid, demised cotwin contributes to the cell‐free DNA (cfDNA) compartment in the maternal circulation.
- Most demised cotwins disappear during the first trimester, but little is known about the dynamics of the resorption process and how long placental tissue of the vanishing twin contributes to the cfDNA pool.

## What does this Report Add?

- We present a case of a dichorionic twin gestation with a demised trisomy‐14 twin and a viable trisomy‐21 twin. Ultrasound showed two separate placentas. Fetal demise occurred between 8 and 9 + 3 weeks of gestation. At 11 + 6 weeks NIPT was indicative of trisomy‐14 and trisomy‐21.
- During the first trimester, remaining cytotrophoblasts of a demised cotwin can contribute to the cfDNA pool, leading to discordant or false‐positive NIPT results for at least 2 weeks and 3 days after demise.

Noninvasive prenatal testing (NIPT) is a test for fetal aneuploidy based on the analysis of cell‐free DNA fragments (cfDNA) isolated from maternal plasma. It is widely used to screen for trisomies 13, 18, and 21 1. The placental DNA fragments are released into the maternal circulation by apoptotic cytotrophoblast cells. Therefore, confined placental mosaicism (CPM) or a vanishing twin can lead to discrepancies between the NIPT result and the fetal genotype. The latter phenomenon refers to the disappearance of an embryo and a gestational sac after documented fetal cardiac activity in both twins of a twin gestation 2. Remaining cytotrophoblasts of a vanishing twin result in the presence of DNA fragments of a third genome in the maternal circulation. When the viable twin has a normal karyotype and the demised twin does not, the NIPT result may be indicative of an aneuploidy and, therefore, “false positive.” Whether an empty sac (a so‐called blighted ovum) can also contribute to cfDNA has not yet been ascertained.

Usually, a vanishing twin becomes invisible at an ultrasound before 13–15 weeks of gestation 2, 3, 4. Little is known about the factors that determine the dynamics of the resorption of the cytotrophoblasts of the demised twin. It is therefore unknown how long these cells can continue to contribute to the cfDNA pool in the maternal circulation and impact NIPT findings. Here, we present a case of a dichorionic twin gestation in which each twin had a different trisomy. This allowed us to distinguish the contributions to the cfDNA pool of each twin, demonstrating that the vanishing twin significantly contributed to the cfDNA pool for at least 2 weeks and 3 days after its demise.

**Figure 1:**
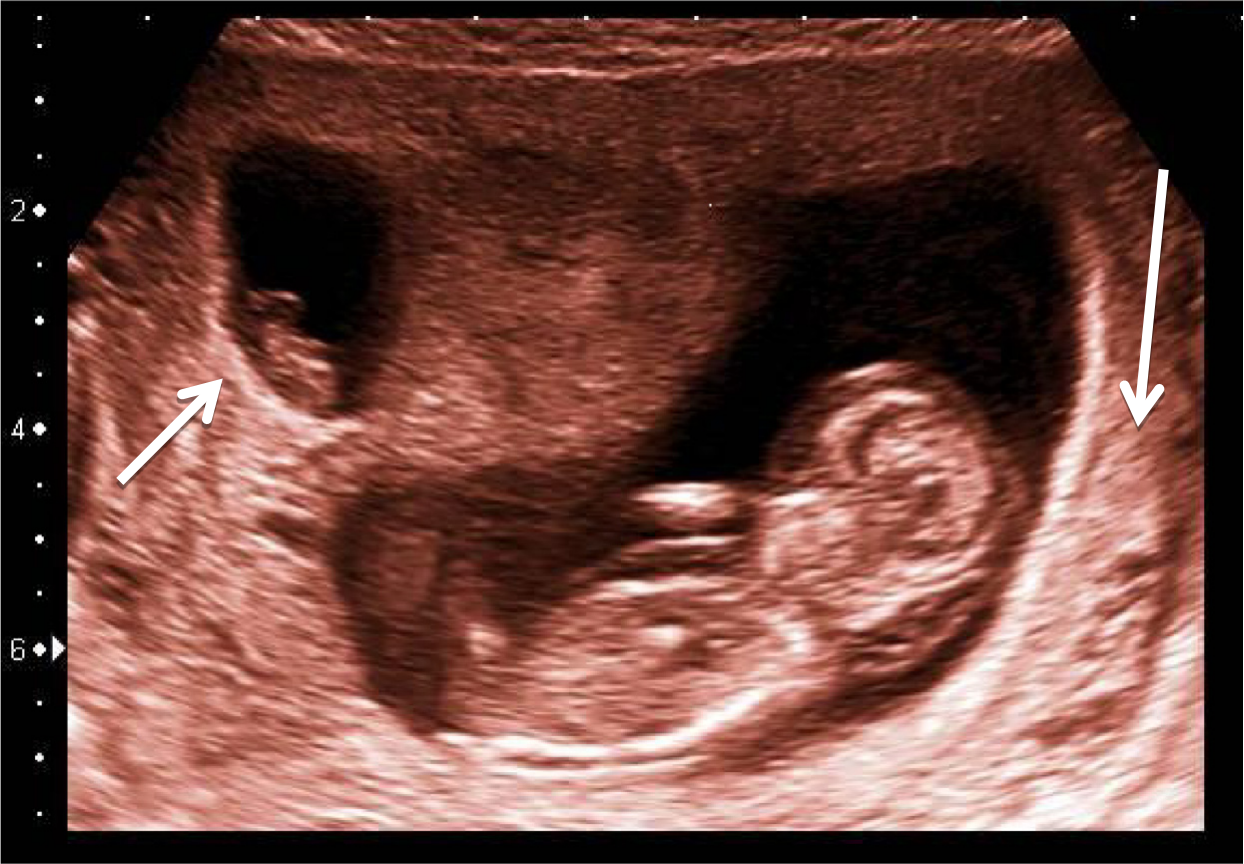
Ultrasound image at 11 + 6 weeks of gestation, immediately prior to sampling of chorionic villi from the viable fetus (long arrow). The chorionic villi of the demised fetus (short arrow) are clearly separated from those of the live fetus.

A 40‐year‐old gravida 2 para 1, with a history of an uneventful pregnancy and spontaneous delivery presented for prenatal counseling at 8 weeks of gestation. The pregnancy had been conceived through intrauterine insemination in a natural cycle. Ultrasonography at 8 weeks showed a viable, dichorionic twin gestation. At 9 + 3 weeks, ultrasound showed the absence of a heartbeat in one fetus and hydrops in the other fetus. The nuchal translucency thickness of the latter fetus was 7.7 mm. Both chorionic masses were separate and a thick intertwine membrane with lambda sign indicated dichorionicity (Fig. 1). Combined first‐trimester testing had already been performed by the midwife, indicating an >1:5 risk for trisomy‐21. Chorionic villus sampling (CVS) was scheduled at 11 + 6 weeks and the patient consented in drawing 9 mL peripheral venous blood for NIPT for research purposes prior to the procedure. Chorionic villi were taken from the viable twin via an abdominal approach and cultured following standard methods. The operator was confident in performing CVS in the viable fetus (Fig. 1). In both the short and long‐term culture a 47,XY,+21 karyotype were seen in 12 and 3 metaphases, respectively. Isolation of cfDNA, next‐ generation sequencing, and bioinformatic analysis for NIPT was performed as described 5. Results were indicative of trisomy‐21 (*z*‐score 18.28), consistent with the karyotype of the viable twin, and, in addition, for trisomy‐14 (*z*‐score 3.02). The cut‐off value for the *z*‐score is 3.00 5. At 12 + 5 weeks, fetal demise was diagnosed in the trisomy‐21 fetus as well. Expulsion was induced by vaginal administration of misoprostol. The fetus, the placenta, and an additional gestational sac were delivered at 13 + 1 weeks and sent to the cytogenetic laboratory. Cell cultures from the fetus in the additional gestational sac were set up for karyotyping. This showed a 47,XY,+14 karyotype in all 16 metaphases investigated, consistent with the NIPT result.

The vanishing twin phenomenon is not uncommon, as shown by several independent estimates of its incidence. In Hungary, almost all pregnant women receive two successive ultrasound examinations during the first trimester. In a cohort of 65,237 pregnancies, a vanishing twin was identified in 0.47% of cases 6. In a Dutch study of 32,222 samples of fetal *RHD* genotyping at 27 weeks of gestation, potential causes of discrepancy (*RHD* positive fetus predicted and RhD negative child born) were investigated in detail. After excluding RhD variants and mistakes due to sample identity and weak PCR signals, the incidence of vanishing twins was estimated at 0.6% of all pregnancies 7. Another estimate can be based on the prevalence of twin pregnancies, which is 1.5–2% in western countries. Because a vanishing twin occurs in about 30% of early recognized twin pregnancies 2, 3, we estimate the incidence at 0.45–0.6% of all pregnancies. Based on these diverse but consistent estimates we conclude that a vanishing twin can be expected in 0.45% to 0.6% of cases when NIPT is used for aneuploidy screening in a general obstetric population. Assuming that the rate of aneuploidies in demised twins is similar to the rate of about 60% in products of conception 8, a false‐positive NIPT result due to a chromosomal anomaly in the vanishing twin is possible in 0.27–0.36% of successive cases. Indeed, in a series of 30,795 consecutive NIPT tests based on genotyping of single‐ nucleotide polymorphisms (SNPs), 130 (0.42%) showed evidence for the presence of a second fetal haplotype. Clinical information was available in 77 cases and in 32 of these a vanishing twin was identified by ultrasound. This corresponds to 0.10% of all tests 4. The estimate of 0.10% is a minimum estimate because vanishing twins most likely also occurred in the 53 cases in which clinical information was lacking. If we assume that the proportion of vanishing twin cases is the same in these 53 cases, a maximum estimate from this study is 0.18% 4.

Documented reports of discrepant NIPT outcomes in pregnancies with a vanishing twin are scarce. Two cases were described by Grömminger et al. 9. In the first case, the demised twin had a 47,XX,+21 karyotype, and fetal heartbeat was absent at 10 weeks of gestation. At 17 + 2 weeks, the demised twin was still visible on ultrasound and NIPT was positive for trisomy‐21 (*z*‐score 13.5). The contribution by the vanishing twin to the cfDNA pool was about 50%. The viable twin showed a normal phenotype at birth and had a normal male karyotype (46,XY). At 38 + 2 weeks of gestation, the vanishing twin, which had developed into a fetus papyraceus, did no longer contribute. There was no evidence for the presence of trisomy‐21 cells in mother, placenta or newborn boy. In their second case 9, blood was sampled for NIPT at 13 + 2 weeks of gestation. A *z*‐score of 3.4 for chromosome 21 and 3.0 for the Y‐chromosome were suggestive of a 47,XY,+21 vanishing twin as the viable twin was normal and of female sex by ultrasonography. The vanishing twin's contribution to the cfDNA pool was estimated at 25%. Apparently, in this case, resorption of the trisomy‐21 twin was almost complete at 13 + 2 weeks. In a case described by Kelley et al. 10, a positive heart‐beat was seen in only one of two sacs at 7 + 4 weeks. NIPT at 13 + 1 weeks was indicative of a 46,XY karyotype, but at delivery a healthy female baby was born with a normal 46,XX karyotype.

In our case, both gestational sacs, one from a 47,XY,+14 and the other from a 47,XY,+21 fetus, continued to contribute to the cfDNA in the maternal circulation for at least 2 weeks and 3 days after the demise of the trisomy‐14 fetus. Our case and those from the literature are summarized in Table 1. We conclude that after fetal demise the cytotrophoblast continues to contribute to the cfDNA pool in the maternal circulation for a variable but potentially considerable time, with 7–8 weeks as the longest period on record for natural cotwin demise 4, 9. In a case of selective fetal reduction at 12 weeks in a triplet pregnancy conceived by in vitro fertilization, DNA fragments of the two reduced embryos were detectable in the maternal circulation 12 weeks later and possibly up to 17 weeks later 11. It is conceivable that cytotrophoblasts from a vanishing twin survive in utero by homing near the spiral arteries of the placenta or by fusing with the placenta of the viable twin 7. Our findings confirm that it is essential to check for the presence of a demised cotwin by detailed ultrasound examination prior to drawing blood for NIPT, as a vanishing twin may occur in 0.45–0.6% of all pregnancies and may lead to discordant outcomes in 0.27–0.36% of consecutive tests. Our results and those of others indicate that a vanishing twin can lead to discordant NIPT results for up to 7–8 weeks after fetal demise.

**Table 1:** Overview of cases with documented contribution by a vanishing twin to the cfDNA in the maternal circulation, leading to a discrepant NIPT result

Study (case) [Reference]	Evidence for presence of a vanishing twin^a^ (weeks+days)	GA at diagnosis of fetal demise^b^ positive NIPT (weeks+days)	GA at NIPT (weeks+days)	Minimal time between fetal demise and false	*z*‐score of trisomy of vanishing twin^c^
Grömminger et al. 2014 (case A) 9	US, VT 47,XX,+21, normal live‐born boy 46,XY	in week 10	17 + 2	~7	13.5
Grömminger et al. 2014 (case B) 9	VT 47,XY,+21, viable twin normal female at US	n.d.	13 + 2	n.d.	3.4
Curnow et al. 2015 (case 1) 4	US, BAF‐plot showing three SNP haplotypes	8 + 0	10 + 3	2 + 3	11.7% ff, no *z*‐score
Curnow et al. 2015 (case 2) 4	US, BAF‐plot showing three SNP haplotypes	7 + 1	10 + 4	3 + 3	4.6% ff, no *z*‐score
Curnow et al. 2015 (case 3) 4	US, BAF‐plot showing three SNP haplotypes	8 + 6	12 + 6	4 + 0	12.8% ff, no *z*‐score
Curnow et al. 2015 (case 4) 4	US, BAF‐plot showing three SNP haplotypes	8 + 0	14 + 7	6 + 7	11.8% ff, no *z*‐score
Curnow et al. 2015 (case 5) 4	US, BAF‐plot showing three SNP haplotypes	7 + 0	15 + 0	8 + 0	8.1% ff, no *z*‐score
Kelley et al. 2016 10	US, VT 46,XY, normal live born girl 46,XX	7 + 4	13 + 1	5 + 4	n.d.
Present case	US, VT 47,XY,+14, viable twin 47,XY,+21	9 + 3	11 + 6	2 + 3	3.02

a US, ultrasound examination; VT, vanishing twin; BAF, biallele frequency; SNP, single‐nucleotide polymorphism.

b Fetal demise is defined by the absence of heartbeat during ultrasound examination; GA, gestational age; n.d., not determined.

c ff = fetal fraction in the total cfDNA pool; in the study by Curnow et al. 4 the NIPT test was based on SNP genotyping and, therefore, *z*‐scores cannot be given.

## Authorship

RH: performed cytogenetic investigations and writing of the manuscript; MGE: performed bioinformatic analysis of NIPT sequencing data; PHAZ: performed sequencing of cfDNA; KDL: was involved in genetic counseling; JH: was involved in genetic counseling; GHS‐B: supervised the performance of NIPT by the laboratory and contributed to the writing of the manuscript; GCMLP‐C: performed pretest counseling, ultrasound investigations, CVS, clinical follow‐up of the patient, and writing of the manuscript.

## Conflict of Interests

The authors declare that there is no conflict of interests regarding the publication of this paper.
